# Evidence for an Adult-Like Type 1-Immunity Phenotype of Vδ1, Vδ2 and Vδ3 T Cells in Ghanaian Children With Repeated Exposure to Malaria

**DOI:** 10.3389/fimmu.2022.807765

**Published:** 2022-02-17

**Authors:** Ximena León-Lara, Tao Yang, Alina Suzann Fichtner, Elena Bruni, Constantin von Kaisenberg, Britta Eiz-Vesper, Daniel Dodoo, Bright Adu, Sarina Ravens

**Affiliations:** ^1^ Institute of Immunology, Hannover Medical School (MHH), Hannover, Germany; ^2^ Department of Obstetrics, Gynecology and Reproductive Medicine, Hannover Medical School (MHH), Hannover, Germany; ^3^ Institute of Transfusion Medicine and Transplant Engineering, Hannover Medical School (MHH), Hannover, Germany; ^4^ Noguchi Memorial Institute for Medical Research, University of Ghana, Accra, Ghana; ^5^ Cluster of Excellence Resolving Infection Susceptibility (RESIST) (EXC 2155), Hannover Medical School (MHH), Hannover, Germany

**Keywords:** *Plasmodium falciparum*, Vδ1, Vδ2, Vδ3, type1- and type3-immunity γδ T cells, childhood

## Abstract

Effector capabilities of γδ T cells are evident in *Plasmodium* infection in young and adult individuals, while children are the most vulnerable groups affected by malaria. Here, we aimed to investigate the age-dependent phenotypic composition of Vδ1^+^, Vδ2^+^, and Vδ3^+^ T cells in children living in endemic malaria areas and how this differs between children that will develop symptomatic and asymptomatic *Plasmodium falciparum* infections. Flow cytometric profiling of naïve and effector peripheral blood γδ T cells was performed in 6 neonates, 10 adults, and 52 children. The study population of young children, living in the same malaria endemic region of Ghana, was monitored for symptomatic *vs* asymptomatic malaria development for up to 42 weeks after peripheral blood sampling at baseline. For the Vδ2^+^ T cell population, there was evidence for an established type 1 effector phenotype, characterized by CD94 and CD16 expression, as early as 1 year of life. This was similar among children diagnosed with symptomatic or asymptomatic malaria. In contrast, the proportion of type 2- and type 3-like Vδ2 T cells declined during early childhood. Furthermore, for Vδ1^+^ and Vδ3^+^ T cells, similar phenotypes of naïve (CD27^+^) and type 1 effector (CD16^+^) cells were observed, while the proportion of CD16^+^ Vδ1^+^ T cells was highest in children with asymptomatic malaria. In summary, we give evidence for an established adult-like γδ T cell compartment in early childhood with similar biology of Vδ1^+^ and Vδ3^+^ T cells. Moreover, the data supports the idea that type 1 effector Vδ1^+^ T cells mediate the acquisition of and can potentially serve as biomarker for natural immunity to *P. falciparum* infections in young individuals from malaria-endemic settings.

## Introduction

Malaria is endemic in large parts of sub-Saharan countries and among the leading cause of death, while children are the most vulnerable group; in 2019, they accounted for 67% (274 000) of all malaria deaths worldwide (1). Children living in endemic areas eventually acquire ‘clinical’ immunity to malaria, with a decline of symptomatic malaria episodes, but they remain vulnerable to harbor parasites as asymptomatic carriers into adulthood (2). The immunologic mechanisms underlying the acquisition of clinical tolerance depend on pleiotropic factors, including the ability to eliminate the parasites, neutralize the parasite virulence factors, and regulate tissue damage triggered by excessive antimalarial responses (3).

One subpopulation of T lymphocytes, γδ T cells, could be essential to early neonatal and childhood protection. They start to develop during early fetal development, show a high functionality at birth and fast expansion upon microbial encounters (4–9). Murine and human γδ T cells are often grouped based on their surface γδ T cell receptor and functionality. It is well received that type 1- and type3-immunity cells are the major functional γδ T cells subsets (10), albeit type 3 cells are less frequent in humans (5). Human type 1 immunity cells comprise Vγ9^+^Vδ2^+^ and Vδ1^+^ T cells that are characterized by cytotoxicity-associated gene expression profiles by being for instance CD27^low^, NK receptor CD94^+^ (mainly Vγ9^+^Vδ2^+^) and CD16^+^. Human type 3 immunity cells express exclusively the Vγ9Vδ2^+^ TCR and genes like *CCR6* and *KLRB1* (encoding CD161) that relate to interleukin-17 production (5).

For human Vγ9^+^Vδ2^+^ T cells pleiotropic roles that range from direct cytotoxicity to antigen-presenting cell capabilities in the early and late stage of Plasmodium parasite control have been assigned (11). In malaria naïve individuals or low transmission settings, Vγ9^+^Vδ2^+^ T cells expand and become rapidly activated upon stimulation with *P. falciparum* antigens during the blood stage of the infection (12–14). Vγ9^+^Vδ2^+^ T cells react to phosphoantigens ((E)-4-hydroxy-3-methyl-but-2-enyl pyrophosphate (HMBPP)) produced by the plasmodial apicoplast and mediate direct killing of intra-erythrocytic parasites by the release of cytotoxic granules and phagocytosis of antibody-coated infected red blood cells (15–17). This is accompanied by IFN-γ and TNF-α release in acute *P. falciparum* infection (17–19) and may even promote pathology (11). However, the functional responsiveness of Vγ9^+^Vδ2^+^ T cells in peripheral blood may decline after chronic exposure and repeated malaria episodes in young individuals, which could be associated with clinical tolerance (20–22). In endemic malaria regions, or after repetitive infections, there is no further peripheral expansion on exposure to the parasite nor malaria-specific production of inflammatory cytokines by this γδ subset (21).

For human Vδ1^+^ T cells, only a handful of studies delineated their role during malaria (23) and reported a focused Vδ1^+^ TCR repertoire and an IFN-γ producing phenotype (24–27). Strikingly, such phenotypes, as well as elevated numbers of circulating Vδ1 T cells, were only evident in individuals from malaria-endemic regions that are regularly exposed to *P. falciparum* and other pathogens (24, 28). High peripheral Vδ1^+^ T cell frequencies might originate from the downregulation of Vγ9^+^Vδ2^+^ T cells during malaria (21) and/or the re-appearance of hepatic Vδ1^+^ T cells upon malaria treatment (24). Similarly, the less-well characterized Vδ3^+^ T cell subset is a minor lymphocyte subset in peripheral blood but is enriched in the liver (29, 30), which may play a role in hepatic stage of malaria. At least murine models highlighted the potential of γδ T cells to modulate liver-stage parasite infections and inflammation that contribute to disease severity (31). Human Vδ3^+^ T cells are more abundant in individuals more frequently exposed to malaria and other infectious diseases (8, 29), while knowledge about their phenotypic distribution is largely lacking. Understanding the acquisition of immunity to malaria among children residing in endemic regions may improve treatment and vaccines for this priority group. Therefore, we aimed to investigate the age-dependent phenotypic composition of Vδ1^+^, Vδ2^+^, and Vδ3^+^ T cells in individuals living in endemic malaria settings. We further aimed to understand potential phenotypic differences among children that develop symptomatic and asymptomatic *Plasmodium falciparum* infections.

## Methods

### Study Population, Sample Collection, and Mononuclear Cell Isolation

Peripheral blood mononuclear cells (PBMCs) were isolated from about 5 ml venous blood samples obtained from children (n=52) living in Asutsuare, Dangme-West District, a malaria-endemic area in the Greater-Accra region of Ghana or from healthy adult donors (n=10) recruited in Legon, also in the Greater-Accra region. The children samples were collected at baseline (enrolment) during a 42-week malaria longitudinal cohort study described in detail elsewhere (32) while the adult samples were from a cross-sectional blood draw protocol. In addition, cord blood mononuclear cells (CBMCs) (n=6) were obtained from uncomplicated, full-term pregnancies delivered at the Hannover Medical School. PBMCs and CBMCs were isolated from fresh EDTA blood samples using Ficoll-Paque density gradient media separation. After isolation, mononuclear cells were frozen in 90% fetal bovine serum (FBS) and 10% DMSO freezing medium. All specimens were stored at -80°C until use.

The study participants were stratified according to their age and malaria status in the following groups: neonates (cord blood) (n=6), control malaria-free children (n=27), control malaria-free adults (n=10) and children with *P. falciparum* malaria (n=25). Malaria was diagnosed by blood slide microscopy. Malaria samples were further stratified as either febrile (> 37.5°C) uncomplicated *P. falciparum* infection (n=7) or asymptomatic parasitemia (n=18). Febrile or uncomplicated malaria was defined as a child being positive for any *P. falciparum* parasitemia by microscopy and fever (axillary temperature > 37.5°C measured or reported) in addition to at least 1 other sign of malaria such as vomiting, diarrhea, or malaise. Asymptomatic parasitemia was defined as children who had no clinical manifestations of malaria despite being microscopy positive at least once during the 42-week follow up period. In addition to not having fever, malaria free status was confirmed as being negative for any parasitemia by both microscopy and polymerase chain reaction (PCR) with specific primers targeting a 276-bp fragment of the 18S rRNA gene of *P. falciparum* as previously described (33). Children under 1 year or over 13 years old or having fever without any detectable *P. falciparum* parasitemia by microscopy at the time of sampling were excluded. Also, adults with malaria infections at time of sampling were excluded from the study.

Children were tested for Cytomegalovirus (CMV) serostatus as described previously using commercially available IgG Western blot kit (34) designed to quantitatively determine anti-CMV IgG antibodies against major CMV proteins (recomLine CMV IgG, Mikrogen, Neuried, Germany). Western Blot was performed according to the manufacturer’s instructions.

Sample collection was done according to the Declaration of Helsinki and the ethics review board at Hannover Medical School (Hannover, Germany) under study numbers 1303-2012 (cord blood donors). Samples collected in Ghana (Africa) were approved by the Institutional Review Board of Noguchi Memorial Institute for Medical Research (NMIMR) of the University of Ghana, Accra, Ghana (NMIMR-IRB CPN 028/07–08 and CPN 109/15–16 amendment 2017). Before sample collection, written informed consent was obtained from all donors (parents or guardians in the case of cord blood and children).

### Flow Cytometry

Thawed mononuclear cells were washed in phosphate-buffer saline (PBS) and treated with DNAse at 0.1mg/ml for 15 min before staining, a maximum of 100.000 PMBCs were stained per sample. Cell suspensions were first stained with a fixable viability dye (Zombie NIR, Biolegend) at room temperature for 15 minutes, and washed with 3% FACS-Buffer. Afterward, cells were stained at room temperature for 20 min with the antibodies listed below using Brillant Stain buffer (BD Bioscience) and fixed with 4.2% paraformaldehyde (BD Bioscience). The acquisition was performed on a Cytek Aurora spectral flow cytometer (Cytek Biosciences).

The following antibodies were used: anti-CD3 AF532 (clone UCHT1; Invitrogen), anti-γδ TCR PE (clone 11F2, Miltenyi Biotec), anti-Vγ9 FITC (clone IMMU 360; Beckman Coulter), anti-Vδ2 PerCP-Vio700 (clone REA771; Miltenyi Biotec), anti-Vδ1 VioGreen (clone REA173; Miltenyi Biotec), anti-Vδ3 (clone D3P11.5B; Beckman Coulter) conjugated with the APC conjugation kit (Abcam), anti-CD27 AF700 (clone O323; BioLegend), anti-CCR7 BV711 (clone G043H7; BioLegend), anti-CD127 BV421 (clone A019D5; BioLegend), anti-PD1 BV605 (clone EH12.2H7; BioLegend), anti-CD16 BUV496 (clone 3G8; BD Bioscience), anti-CD94 PE-Cy7 (clone DX22; BioLegend), anti-CCR6 BV785 (clone G034E3; BioLegend), anti-CD161 APC-Cy7 (clone HP-3G10; BioLegend), anti-CCR4 BV650 (clone 1G1; BD Bioscience) and anti-CD45RA PE-Cy5 (clone HI100; eBioscience).

### Computational Analysis Of Flow Cytometry Data

Flow cytometry (FACS) data were analyzed by FlowJoTM v10.7.2 software and R v4.0.3 using the Spectre R package (35), with instructions and source code provided at https://github.com/ImmuneDynamics/spectre. Briefly, TCR γδ+ population was gated in FlowJoTM v10.7.2 software and exported as raw value CSV files to R v4.0.3, resulting in a median of 1115 (108-28 849) γδ T cells per sample. Next, Arcsinh transformation was performed on the data in R using a co-factor of 5 000 to redistribute the data on a linear scale and compress low-end values near zero. The FlowSOM algorithm (36) was then run on the merged dataset using all γδ TCR^+^ cells per sample (total 176 283 γδ T cells) to cluster the data, where every cell is assigned to a specific cluster and meta-cluster. Subsequently, the data were downsampled and analyzed by the dimensionality reduction algorithm Uniform Manifold Approximation and Projection (UMAP) (McInnes, Healy, Melville, 2018) for cellular visualization; 60 000 γδTCR^+^ cells were visualized on the UMAP.

### Statistical Analysis

Statistical analyses were performed with R v4.0.3. Comparisons between multiple groups were performed using ANOVA with Tuckey *post hoc* test.

## Results

### Flow Cytometric Analysis of Peripheral Blood γδ T Cells Reveals Similar Phenotypes of Vδ1 and Vδ3 T Cells in Young Individuals

First, we aimed to investigate the phenotypic composition of Vδ1^+^, Vδ2^+^, and Vδ3^+^ T cells in children from malaria-endemic areas. In this sense, γδ T cells were subjected to flow cytometric analysis with antibodies specific for Vδ1^+^, Vδ2^+^, Vδ3^+^ and Vγ9^+^ T cell receptors (TCRs). At the same time, antibodies against CD27, CD45RA, CCR7, and CD127 were used to identify naïve and central memory cells; CCR4 for type 2-immunity γδ T cells; CCR6 and CD161 for innate type 3-immunity γδ T cells; and CD94, CD16, and PD1 for cytotoxic type 1 effector γδ T cells (5).

**Figure 1:**
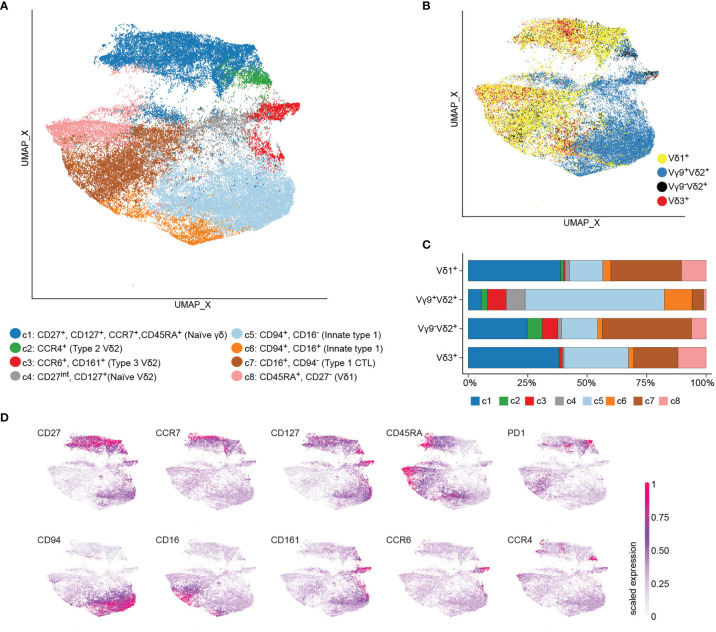
Flow cytometry data reveals heterogeneity across γδ T cell subsets. Peripheral blood mononuclear cells of children with malaria (n=25), non-infected children (n=27) from Ghana, Ghanaian non-infected adults (n=10), and European cord blood donors (n=6) were stained with a 17-color antibodies panel to delimit γδ T cell subsets. Flow cytometry data of γδ T cells were subjected to unsupervised clustering, down sampled and visualized by UMAP, where each point represents a single cell. **(A)** UMAP color-coded by cluster. **(B)** Single cells colored-code by being Vδ1^+^, Vγ9^+^Vδ2^+^, Vγ9^-^Vδ2^+^ or Vδ3^+^. **(C)** The proportion of each cluster was calculated for the total number of Vδ1^+^, Vγ9^+^Vδ2^+^, Vγ9^-^Vδ2^+^ or Vδ3^+^ T cells of all included subjects. **(D)** Expression values of the ten surface markers were scaled to a range between 0 and 1 and projected on UMAP.

In total, we profiled 68 peripheral blood γδ T cell samples from six European neonates, fifty-two Ghanian children, and ten Ghanian adults (**Table 1**). The neonatal samples included were obtained from six European cord blood donors and primarily served as staining controls for naive and type 3 effector phenotypes. Then we performed an unsupervised clustering, using the total 176.283 TCR γδ^+^ cells from the 68 samples, based on expression levels of the ten surface markers. We identified eight clusters (c1 – c8) as projected in UMAP (**Figure 1A**). All three donor groups contributed to all identified eight clusters, albeit with visible quantitative differences. TCR γδ^+^ cells from neonatal samples contributed less than one percent to each of the clusters c5 to c8 (**Supplementary Figures 1 A–C**). Overlying TCR V-gene usage information gives evidence that Vδ1^+^ and Vγ9^+^Vδ2^+^ T cells clearly separate and that Vδ3^+^ T cells clustered together with Vδ1^+^ T cells (**Figure 1B**). No cluster was exclusive to a specific γδ T cell subset, however the proportion of each cluster varied among Vγ9^+^Vδ2^+^ and Vδ1/3^+^ cells. Notably, clusters c2, c3 and c4 were less than four percent of Vδ1 and two percent of Vδ3. In the Vγ9^+^Vδ2^+^ T cell compartment cluster c8 was less than one percent (**Figure 1C**). Next, we assigned the identified clusters to naïve and effector phenotypes based on the differential expression of ten surface markers (**Figures 1A, D**). There is a naïve and mostly cord blood-derived γδ T cell cluster (c1), composed of Vδ1^+^, Vδ2^+^ and Vδ3^+^ cells, defined as CD27^+^ and CD127^+^ (c1) with variable expression of CCR7, CD45RA, and PD1 on Vδ1^+^ T cells. For Vγ9^+^Vδ2^+^ T cells a CD27^int^/CD127^+^ naïve fraction (c4), a type 2-related CCR4^+^ cluster (c2) and a type 3-related CCR6^+^ and CD161^+^ cluster (c3) were identified. The majority of Vγ9^+^Vδ2^+^ T cells were distinguished by high CD94 expression (c5), highlighting the innate cytotoxic properties of this subset, with some also being CD16^+^ (c6). A large fraction of Vδ1^+^ T cells showed a CD16^+^ type 1 effector phenotype (c7) and evidence for CD27^neg^, CCR7^neg^, CD45RA^+^ (c8), accounting for 29.6% and 10.5% of all cells, respectively. Notably, few Vδ1^+^ T cells were positive for the NK receptor CD94. Interestingly, the less well-studied Vδ3^+^ T cell compartment clustered closed to the Vδ1^+^ T cells by either displaying a naïve (c1) or CD16^+^ cytotoxic effector phenotype (c7). Together, the detailed flow cytometry analysis revealed high heterogeneity across different γδ T cell compartments in young individuals, with similar effector phenotypes of Vδ1^+^ and Vδ3^+^ T cells.

**Table 1:** Demographic characteristics of the patients.

	Control malaria-free (n=43)	Asymptomatic parasitemia (n=18)	Febrile malaria (n=7)
Female- no. (%)*	17/27 (63)	12 (66)	6 (86)
Age distribution- no.(%)			
Neonates (cord blood)	6 (14)	–	–
Young children (1-6 yrs)	12 (28)	6 (33)	5 (71)
Older children (7-11 yrs)	15 (35)	12 (67)	2 (29)
Adults	10 (23)	–	–
Median age (min.-max.)*	7 (1-11)	8 (2-11)	5 (3-8)
CMV serology*			
Positive IgG (%)	22/27 (81)	14 (78)	6 (86)
Negative IgG (%)	1/27 (4)	2 (11)	
Unknown	4/27 (15)	2 (11)	1 (14)

*Information presented for children only.

### Human γδ T Cell Display Age-Dependent Heterogeneity Across γδ T Cell Subsets in Children Living in Malaria Transmission Settings

**Figure 2:**
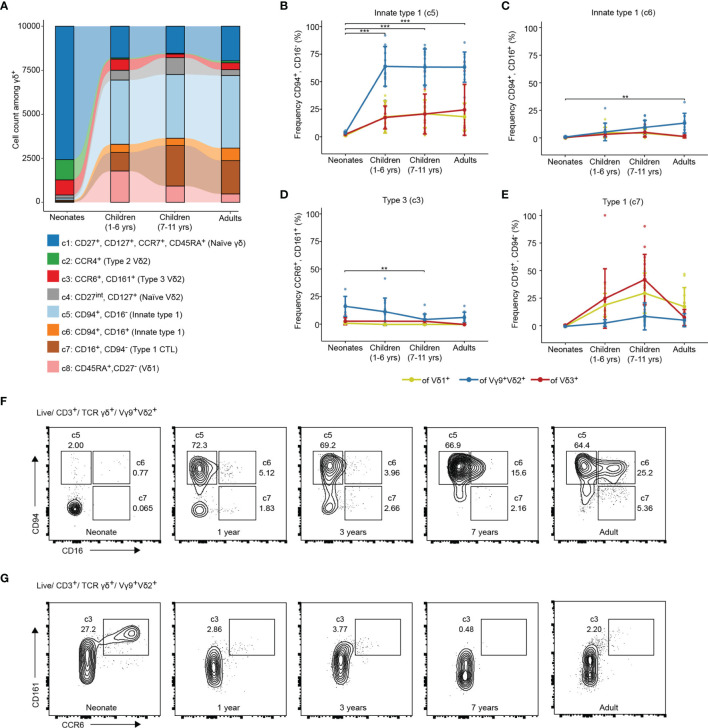
γδ T cells show adult-like type1-phenotype in young and older children living in endemic malaria settings. **(A)** Cell count of the clusters identified from total γδ T cells of newborns (cord blood, n=6), young children (1-6 year old, n=12), older children (7-11 years old, n=15) and adults (n=10). Frequencies of **(B)** CD94^+^/CD16^-^; **(C)** CD94^+^/CD16^+^; **(D)** CCR6^+^/CD161^+^ or **(E)** CD16^+^/CD94^-^ γδ T effectors of the total Vδ1^+^, Vγ9^+^Vδ2^+^ or Vδ3^+^ T cells in newborns (cord blood, n=6), young children (1-6 year old, n=12), older children (7-11 years old, n=15) and adults (n=10). Error bars indicate mean ± SD. Results of Vγ9^+^Vδ2^+^ comparisons by Tukey *post-hoc* test after ANOVA are shown. **p < 0.01, ***p < 0.001. Flow cytometric plots of **(F)** CD94/CD16 and **(G)** CD161/CCR6 on Vγ9^+^Vδ2^+^ T cells in representative samples from a neonate, children without malaria aged 1, 2 and 7 years and one adult.

Next, a more nuanced view about the age-dependent distributions of γδ T cell effector phenotypes and how this is related to TCR usage was obtained. Donors without evidence of malaria (control malaria-free group) were divided into newborns (cord blood, n=6), young (1 – 6 year old, n=12) and older (7 – 11 years old, n=15) children, as well as adults (n=10) (**Table 1**). Age-dependent changes are visible in the overall contribution of donor groups to identified cell clusters (**Figure 2A**). Moreover, the proportion of γδ T cells among total CD3^+^ T cells is significantly higher in Ghanaian young individuals as compared to European neonates (cord blood) (median 2% vs. 15%, p =0.001) (**Supplementary Figure 2A**). Those are primarily Vγ9^+^Vδ2^+^ or Vδ1^+^ T cells (**Supplementary Figure 2B**). Children and adult Vγ9^+^Vδ2^+^ T cells are characterized by being CD94 positive, correlating to c5 with an increase of CD16 expression (c6) in older children and adults (**Figures 2B, C, F**). A fraction of Vδ1^+^ and Vδ3^+^ T cells was also CD94^+^ in these age groups (**Figure 2B**). In contrast, the abundance of type 2 (c2) and type 3 (c3) effector phenotypes, being Vγ9^+^Vδ2^+^, was largely decreased in peripheral blood samples of all children and adults as compared to neonatal cord blood (**Figures 2D, G** and **Supplementary Figures 2C, E**). Vδ1^+^ and Vδ3^+^ T cells showed increase of CD16^+^ cytotoxic effector phenotype during child- and adulthood (**Figure 2E**). *Vice versa*, acquisition of type 1 effector phenotypes (all subsets) were reflected in lower frequencies of naïve γδ T cells (each subset) in young individuals as compared to neonatal cord blood samples (**Supplementary Figure 2D**). In sum, each of the subsets, namely Vγ9^+^Vδ2^+^, Vδ1^+^ or Vδ3^+^ T cells had similar phenotypes in Ghanaian children and adults that largely differed from mostly naive neonatal cord blood cells. Thereby a high abundance of CD94^+^ innate type 1 effector Vγ9^+^Vδ2^+^ T cell with partial CD16 expression in older individuals was evident.

### Vδ1^+^, but Not Vδ3^+^ T Cells Show Increased CD16 Expression in Children Diagnosed With Asymptomatic Malaria

**Figure 3:**
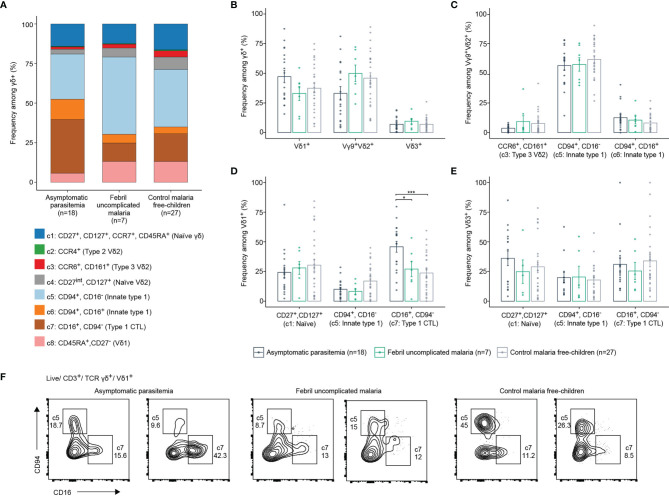
γδ T cell effectors in malaria-free and malaria-positive children. **(A)** Frequency of each identified cluster of total γδ T cells in children with asymptomatic parasitemia (n=18), febrile uncomplicated malaria (n=7) or control malaria free-children (n=27). **(B)** Comparison of Vδ1+, Vγ9^+^Vδ2^+^ or Vδ3^+^ frequencies of γδ T cells according to malaria status of children. **(C)** Comparison of CCR6^+^/CD161^+^, CD94^+^/CD16^-^ or CD94^+^/CD16^-^ frequencies among Vγ9^+^Vδ2^+^ according to malaria status of children. **(D)** Comparison of CD27^+^/CD127^+^, CD94^+^/CD16^-^ or CD16^+^/CD94^-^ frequencies among Vδ1^+^ or **(E)** Vδ3^+^. Error bars indicate mean ± SD, data analysed by ANOVA and Tukey *post-hoc* test. *p < 0.05, ***p < 0.001. **(F)** Flow cytometric plots of CD94/CD16 on Vδ1^+^ T cells in representative samples from two asymptomatic parasitemia, two febrile uncomplicated malaria or two control malaria free-children.

Next, we investigated if γδ T cell phenotypes within the study population of young children relates to symptomatic *vs* asymptomatic *P. falciparum* infection. Thus, we focused on sample analysis of the young and older children, which all live in the same endemic region of Ghana. All peripheral blood samples analyzed by flow cytometry were collected at baseline. Children were monitored for 42 weeks and subsequently stratified in either asymptomatic parasitemia, febrile malaria or malaria-free group (**Figure 3A** and **Table 1**). All clusters identified of the total γδ T cells analyzed by flow cytometry are present in all respective three groups, while abundance of c5-7 γδ T cells slightly differed in asymptomatic malaria children (**Figure 3A** and **Supplementary Figure 3A**). Moreover, Vδ1^+^, Vγ9^+^Vδ2^+^ and Vδ3^+^ T cell frequencies of CD3^+^ or γδTCR^+^ T cells were similar among all three groups (**Figure 3B** and **Supplementary Figure 3B**). For Vγ9^+^Vδ2^+^ T cells, control, asymptomatic and febrile malaria groups showed no significant differences in the abundance of CCR6^+^ type 3, CD94^+^ innate type 1, and CD94^+^, CD16^+^ double-positive type 1 effector phenotypes (**Figure 3C**). For Vδ1^+^ T cells a significant increase of CD16^+^ CD94^neg^ type 1 CTL effector phenotypes, representing c7, was observed for the asymptomatic malaria group (**Figures 3D, F**). The less-well studied Vδ3^+^ T cells displayed a similar abundance of naïve, innate-type 1 or CTL effector phenotypes that did not differ among the respective three patient groups (**Figure 3E**). In sum, the flow cytometric analysis reported no major phenotypic changes of Vγ9^+^Vδ2^+^ and Vδ3^+^ T cells among the three groups based on malaria status and an increase of CD16^+^ Vδ1^+^ T cells in children diagnosed with asymptomatic malaria.

## Discussion

In this study γδ T cells were profiled by flow cytometry in Ghanaian children, and compared to European neonates and Ghanian adults, to understand how γδ T cell populations are impacted by age and malaria infection. For Vγ9Vδ2^+^ T cells, we focused on innate-like type 1, type 2 and type 3 effector phenotypes and found evidence for increased CD94 expression on the majority of Vγ9Vδ2^+^ T cells in 1 – 11 years old children (Ghana) as compared to neonates (Europe). Similar to previous reports, this distribution of CD94^+^ and low abundance of naïve Vγ9Vδ2^+^ T cells was already evident at 1 year of life (37–39). Thereby a gradual increase of CD94 might be evident during the early life period (9). We further observed reduced frequencies, but no loss of blood CCR6^+^/CD161^+^ Vγ9Vδ2^+^ T cells that were recently described as fetal-thymus derived type3-immunity γδ T cells (5). It remains unknown if this is due to homing of CCR6^+^ γδ T cells to defined anatomic locations or a better proliferative capacity of innate type 1 Vγ9Vδ2^+^ T cells upon birth. Similarly, these speculations hold true for CCR4^+^ Vγ9Vδ2^+^ T cells (type 2). To receive a better understanding of this subsets, the establishment of longitudinal cohorts or the examination of cord blood γδ T cells in neonates from malaria endemic regions are necessary. In the children from endemic transmission settings analysed in this study, the abundance of innate type 1 Vγ9Vδ2^+^ T cells, defined by CD94 and partial CD16 expression was similar in the young and older individuals. This was independent of the susceptibility towards symptomatic malaria, examined in the follow-up period. Thus, it could well be that frequent malaria exposure during early infancy may have had already induced progressive unresponsiveness of blood Vγ9Vδ2^+^ T cells, further correlating with clinical tolerance (18, 21, 22). In line with this idea, it seems that asymptomatic malaria might associate to the here observed slightly lower Vγ9Vδ2 T cell frequencies.

For Vδ1^+^ T cells oligoclonal expansions that often dominate the malaria γδ T cell response by IL-10 and IFN-γ secretion are evident in children and adults living in malaria-endemic areas (24, 25). Along that line, repeated malaria infection, including the exposition to *P.falciparum*-derived antigens, drives the TCR-mediated clonal selection of effector Vδ1^+^ T cells (27). Here we report that the overall frequencies of Vδ1^+^ T cells displaying a CD16^+^ type 1 effector phenotype were increased in asymptomatic malaria children compared to febrile malaria or control groups. However, one limitation of this study population is that only a small number of children became malaria positive during the follow-up period. Moreover, we cannot exclude previous exposure to *Plasmodium* or Epstein-Barr-Virus, for which Vδ1^+^ T cell responses are well implied (40–44). In particular, Cytomegalovirus (CMV) infection may also impact the CD16 expression of Vδ1^+^ T cells (38, 45). As the large majority of children were CMV seropositive the here observed differences among control and malaria positive individuals were not due to CMV. Overall, we suggest that Vδ1^+^ T cells contribute to and/or indicate the naturally acquired immunity against *P. falciparum*, and this might be enhanced by the pathogen-induced maturation of the Vδ1^+^ T cell population (23). Future studies employing larger pediatric patient cohorts, focusing on the relationship of adaptive-like expansion of Vδ1^+^ T cells, CMV serostatus, CD16 expression and asymptomatic *vs* symptomatic malaria, would allow to design γδ T cell based biomarkers to indicate tolerance acquisition towards symptomatic parasitemia.

Another focus was set on the characterization of Vδ3^+^ T cells assigned to have individual, clonal expansion in various settings (8, 30, 46) and capability to recognize CD1d (29). Here we show that Vδ1^+^ and Vδ3^+^ T cells clustered close together by displaying similar surface phenotypes, albeit Vδ3^+^ T cells had higher frequencies of CD94^+^ and CCR6^+^ cells. With regard to *P. falciparum* infections, Vδ3^+^ T cells showed strikingly similar phenotypes among children with asymptomatic and febrile malaria. Longitudinal analysis would be necessary to examine the impact of *P. falciparum* exposure on their functional maturation, including long-term consequences.

Together with TCR repertoire analysis (8, 30) the phenotyping data gives evidence for a similar biology of Vδ1^+^ and Vδ3^+^ T cells. The phenotypic characterization of all main human γδ T cell subsets in young individuals emphasized their potential differential roles and how this relates to age and malaria exposure. The small number of malaria-infected children analyzed may have biased the outcome of this study. To obtain a full picture of how malaria and other infectious diseases impact the role of γδ T cells in the acquisition of malaria tolerance, a longitudinal analysis of a large cohort of unexposed young individuals in endemic settings would be important.

## Data Availability Statement

The original contributions presented in the study are included in the article/**Supplementary Materials**. Further inquiries can be directed to the corresponding authors.

## Ethics Statement

The studies involving human participants were reviewed and approved by the Ethics review board of Hannover Medical School (Hannover, Germany) under study numbers 1303-2012 and Institutional Review Board of Noguchi Memorial Institute for Medical Research (NMIMR) of the University of Ghana, Accra, Ghana (NMIMR-IRB CPN 028/07–08 and CPN 109/15–16 amendment 2017). Written informed consent to participate in this study was provided by the participants’ legal guardian/next of kin.

## Author Contributions

XL, AF, BA, and SR designed the study and experiments XL, AF, and BE-V conducted experiments. XL analyzed data. TY conducted bioinformatic analysis. EB contributed in data analysis. CK, DD, and BA recruited and coordinated study participants. XL and SR wrote the manuscript. All authors contributed to the article and approved the submitted version.

## Funding

This study was supported by the German Research Foundation Deutsche Forschungsgemeinschaft (DFG) under Germany’s Excellence Strategy, EXC 2155 “RESIST” Project ID 390874280 to SR, CRC900 Project ID 158989968 to SR and BE-V, the Ministry of Foreign Affairs of Denmark, Project ID 14-P01-GHA to DD and BA and European and Developing Countries Clinical Trials Partnership Project ID TA.2007.40200.012 to DD. XL was supported by the Hannover Biomedical Research School (HBRS) and the Center for Infection Biology (ZIB).

## Conflict of Interest

The authors declare that the research was conducted in the absence of any commercial or financial relationships that could be construed as a potential conflict of interest.

## Publisher’s Note

All claims expressed in this article are solely those of the authors and do not necessarily represent those of their affiliated organizations, or those of the publisher, the editors and the reviewers. Any product that may be evaluated in this article, or claim that may be made by its manufacturer, is not guaranteed or endorsed by the publisher.

## Supplementary Material

Supplementary Figure 1 Contribution to the identified clusters by age group. **(A)** Single cells visualized on UMAP color-code by age group in newborns (cord blood, n=6), children (n=52) and adults (n=10). **(B)** γδ T cell count contribution of newborns (cord blood, n=6), children (n=52), and adults (n=10) to each identified cluster in cell count and **(C)** frequency.

Supplementary Figure 2 Age-dependent distribution of γδ T cell subsets. **(A)** Frequencies of γδ T cells in newborns (cord blood, n=6), young children (1-6 year old, n=12), older children (7-11 years old, n=15), and adults (n=10) without malaria. **(B)** The frequency of Vδ1^+^, Vγ9^+^Vδ2^+^ or Vδ3^+^ of CD3^+^ γδ T cells per age group in malaria-free donors. **(C)** Frequencies of CCR4^+^ and **(D)** CD27^int^/CD127^+^ of the total Vδ1^+^, Vγ9^+^Vδ2^+^ or Vδ3^+^ T cells. Data was analysed by ANOVA and Turkey *post-hoc* test, figure C shows the results of Vγ9^+^Vδ2^+^ T cell comparisons. Error bars indicate mean + SD. *p < 0.05, **p < 0.01, ***p < 0.001. **(E)** Flow cytometric plot CCR4 on Vγ9^+^Vδ2^+^ T cells in representative samples from a neonate, children without malaria aged 1, 2 and 7 years and one adult.

Supplementary Figure 3 γδ T cell effectors in malaria-free and malaria-positive children. **(A)** Single cells visualized on UMAP color- code by malaria status in either asymptomatic parasitemia (n=18), febrile uncomplicated malaria (n=7) or control malaria free-children (n=27). **(B)** Comparison of Vδ1^+^, Vγ9^+^Vδ2^+^ or Vδ3^+^ T cell frequencies of CD3^+^ T cells according to malaria status of children.

## References

[1] World Malaria Report 2019. In: World Malaria Report 2019. World Health Organization.

[2] Tran TM Li S Doumbo S Doumtabe D Huang C-Y Dia S. An Intensive Longitudinal Cohort Study of Malian Children and Adults Reveals No Evidence of Acquired Immunity to Plasmodium Falciparum Infection. Clin Infect Dis (2013) 57:40–7. doi: 10.1093/cid/cit174 PMC3669526 23487390

[3] Galatas B Bassat Q Mayor A. Malaria Parasites in the Asymptomatic: Looking for the Hay in the Haystack. Trends Parasitol (2016) 32:296–308. doi: 10.1016/J.PT.2015.11.015 26708404

[4] Vermijlen D Brouwer M Donner C Liesnard C Tackoen M Van Rysselberge M. Human Cytomegalovirus Elicits Fetal Gammadelta T Cell Responses *In Utero* . J Exp Med (2010) 207:807–21. doi: 10.1084/jem.20090348 PMC2856038 20368575

[5] Tan L Fichtner AS Bruni E Odak I Sandrock I Bubke A. A Fetal Wave of Human Type 3 Effector γδ Cells With Restricted TCR Diversity Persists Into Adulthood. Sci Immunol (2021) 6:eabf0125. doi: 10.1126/sciimmunol.abf0125 33893173

[6] Dimova T Brouwer M Gosselin F Tassignon J Leo O Donner C. Effector Vγ9vδ2 T Cells Dominate the Human Fetal γδ T-Cell Repertoire. Proc Natl Acad Sci (2015) 112:E556–65. doi: 10.1073/pnas.1412058112 PMC4330771 25617367

[7] Gibbons DL Haque SFY Silberzahn T Hamilton K Langford C Ellis P. Neonates Harbour Highly Active γδ T Cells With Selective Impairments in Preterm Infants. Eur J Immunol (2009) 39:1794–806. doi: 10.1002/eji.200939222 19544311

[8] Ravens S Fichtner AS Willers M Torkornoo D Pirr S Schöning J. Microbial Exposure Drives Polyclonal Expansion of Innate γδ T Cells Immediately After Birth. Proc Natl Acad Sci USA (2020) 117:18649–60. doi: 10.1073/pnas.1922588117 PMC7414158 32690687

[9] Papadopoulou M Dimova T Shey M Briel L Veldtsman H Khomba N. Fetal Public Vγ9vδ2 T Cells Expand and Gain Potent Cytotoxic Functions Early After Birth. Proc Natl Acad Sci USA (2020) 117:18638–48. doi: 10.1073/pnas.1922595117 PMC7414170 32665435

[10] Ribot JC Lopes N Silva-Santos B. γδ T Cells in Tissue Physiology and Surveillance. Nat Rev Immunol (2021) 21:221–32. doi: 10.1038/s41577-020-00452-4 33057185

[11] Howard J Zaidi I Loizon S Mercereau-Puijalon O Déchanet-Merville J Mamani-Matsuda M. Human Vγ9vδ2 T Lymphocytes in the Immune Response to P. Falciparum Infection. Front Immunol (2018) 9:2760. doi: 10.3389/fimmu.2018.02760 30538708 PMC6277687

[12] Costa G Loizon S Guenot M Mocan I Halary F de Saint-Basile G. Control of Plasmodium Falciparum Erythrocytic Cycle: γδ T Cells Target the Red Blood Cell-Invasive Merozoites. Blood (2011) 118:6952–62. doi: 10.1182/blood-2011-08-376111 22045985

[13] Hviid L Kurtzhals JAL Dodoo D Rodrigues O Rønn A Commey JO. The Gamma/Delta T-Cell Response to Plasmodium Falciparum Malaria in a Population in Which Malaria Is Endemic. Infect Immun (1996) 64:4359–62. doi: 10.1128/iai.64.10.4359-4362.1996 PMC174380 8926112

[14] Ho M Webster HK Tongtawe P Pattanapanyasat K Weidanz WP. Increased γδ T Cells in Acute Plasmodium Falciparum Malaria. Immunol Lett (1990) 25:139–41. doi: 10.1016/0165-2478(90)90105-Y 2149360

[15] Farouk SE Mincheva-Nilsson L Krensky AM Dieli F Troye-Blomberg M. γ δ T Cells Inhibit*In Vitro* Growth of the Asexual Blood Stages Ofplasmodium Falciparum by a Granule Exocytosis-Dependent Cytotoxic Pathway That Requires Granulysin. Eur J Immunol (2004) 34:2248–56. doi: 10.1002/eji.200424861 15259022

[16] Junqueira C Polidoro RB Castro G Absalon S Liang Z Sen Santara S. γδ T Cells Suppress Plasmodium Falciparum Blood-Stage Infection by Direct Killing and Phagocytosis. Nat Immunol (2021) 22:347–57. doi: 10.1038/s41590-020-00847-4 PMC7906917 33432229

[17] Hernández-Castañeda MA Happ K Cattalani F Wallimann A Blanchard M Fellay I. γδ T Cells Kill Plasmodium Falciparum in a Granzyme- and Granulysin-Dependent Mechanism During the Late Blood Stage. J Immunol (2020) 204:1798–809. doi: 10.4049/jimmunol.1900725 PMC7086388 32066596

[18] Jagannathan P Lutwama F Boyle MJ Nankya F Farrington LA McIntyre TI. Vδ2+ T Cell Response to Malaria Correlates With Protection From Infection But Is Attenuated With Repeated Exposure. Sci Rep (2017) 7:11487. doi: 10.1038/s41598-017-10624-3 28904345 PMC5597587

[19] D’Ombrain M Hansen DS Simpson KM Schofileld L. Gammadelta-T Cells Expressing NK Receptors Predominate Over NK Cells and Conventional T Cells in the Innate IFN-Gamma Response to Plasmodium Falciparum Malaria. Eur J Immunol (2007) 37:1864–73. doi: 10.1002/EJI.200636889 17557374

[20] Goodier M Krause-Jauer M Sanni A Massougbodji A Sadeler B-C Mitchell GH. γδ T Cells in the Peripheral Blood of Individuals From an Area of Holoendemic Plasmodium Falciparum Transmission. Trans R Soc Trop Med Hyg (1993) 87:692–6. doi: 10.1016/0035-9203(93)90299-6 8296383

[21] Jagannathan P Kim CC Greenhouse B Nankya F Bowen K Eccles-James I. Loss and Dysfunction of Vδ2+γδ T Cells Are Associated With Clinical Tolerance to Malaria. Sci Transl Med (2014) 6:251ra117. doi: 10.1126/SCITRANSLMED.3009793 PMC4198150 25163477

[22] Farrington LA Jagannathan P McIntyre TI Vance HM Bowen K Boyle MJ. Frequent Malaria Drives Progressive Vδ2 T-Cell Loss, Dysfunction, and CD16 Up-Regulation During Early Childhood. J Infect Dis (2016) 213:1483–90. doi: 10.1093/infdis/jiv600 PMC4813738 26667315

[23] Hviid L Smith-Togobo C Willcox BE. Human Vδ1+ T Cells in the Immune Response to Plasmodium Falciparum Infection. Front Immunol (2019) 10:259. doi: 10.3389/fimmu.2019.00259 30837999 PMC6382743

[24] Hviid L Kurtzhals JAL Adabayeri V Loizon S Kemp K Goka BQ. Perturbation and Proinflammatory Type Activation of Vδ1+ γδ T Cells in African Children Withplasmodium Falciparum Malaria. Infect Immun (2001) 69:3190–6. doi: 10.1128/IAI.69.5.3190-3196.2001 PMC98276 11292740

[25] Taniguchi T Md Mannoor K Nonaka D Toma H Li C Narita M. Watanabe H. A Unique Subset of γδ T Cells Expands and Produces IL-10 in Patients With Naturally Acquired Immunity Against Falciparum Malaria. Front Microbiol (2017) 8:1288. doi: 10.3389/fmicb.2017.01288 28769886 PMC5515829

[26] Worku S Björkman A Troye-Blomberg M Jemaneh L Färnert A Christensson B. Lymphocyte Activation and Subset Redistribution in the Peripheral Blood in Acute Malaria Illness: Distinct γδ+ T Cell Patterns in Plasmodium Falciparum and P. Vivax Infections. Clin Exp Immunol (1997) 108:34–41. doi: 10.1046/j.1365-2249.1997.d01-981.x 9097908 PMC1904634

[27] von Borstel A Chevour P Arsovski D Krol JMM Howson LJ Berry AA. Repeated Plasmodium Falciparum Infection in Humans Drives the Clonal Expansion of an Adaptive γδ T Cell Repertoire. Sci Transl Med (2021) 13:eabe7430. doi: 10.1126/scitranslmed.abe7430 34851691 PMC9291638

[28] Hviid L Akanmori BD Loizon S Kurtzhals JA Ricke CH Lim A. High Frequency of Circulating Gamma Delta T Cells With Dominance of the V(Delta)1 Subset in a Healthy Population. Int Immunol (2000) 12:797–805. doi: 10.1093/intimm/12.6.797 10837407

[29] Mangan BA Dunne MR O’Reilly VP Dunne PJ Exley MA O’Shea D. Cutting Edge: CD1d Restriction and Th1/Th2/Th17 Cytokine Secretion by Human Vδ3 T Cells. J Immunol (2013) 191:30–4. doi: 10.4049/jimmunol.1300121 PMC3721026 23740951

[30] Hunter S Willcox CR Davey MS Kasatskaya SA Jeffery HC Chudakov DM. Human Liver Infiltrating γδ T Cells Are Composed of Clonally Expanded Circulating and Tissue-Resident Populations. J Hepatol (2018) 69:654–65. doi: 10.1016/j.jhep.2018.05.007 PMC6089840 29758330

[31] Ribot JC Neres R Zuzarte-Luís V Gomes AQ Mancio-Silva L Mensurado S. γδ-T Cells Promote IFN-γ-Dependent Plasmodium Pathogenesis Upon Liver-Stage Infection. Proc Natl Acad Sci USA (2019) 116:9979–88. doi: 10.1073/pnas.1814440116 PMC6525508 31028144

[32] Adu B Dodoo D Adukpo S Hedley PL Arthur FKN Gerds TA. Fc Gamma Receptor IIIB (Fcγriiib) Polymorphisms Are Associated With Clinical Malaria in Ghanaian Children. PloS One (2012) 7:e46197. doi: 10.1371/journal.pone.0046197 23049979 PMC3458101

[33] Adu B Issahaque Q-A Sarkodie-Addo T Kumordjie S Kyei-Baafour E Sinclear CK. Microscopic and Submicroscopic Asymptomatic Plasmodium Falciparum Infections in Ghanaian Children and Protection Against Febrile Malaria. Infect Immun (2020) 88:e00125–20. doi: 10.1128/IAI.00125-20 PMC7504941 32719157

[34] Sukdolak C Tischer S Dieks D Figueiredo C Goudeva L Heuft H-G. CMV-, EBV- and ADV-Specific T Cell Immunity: Screening and Monitoring of Potential Third-Party Donors to Improve Post-Transplantation Outcome. Biol Blood Marrow Transplant (2013) 19:1480–92. doi: 10.1016/j.bbmt.2013.07.015 23891747

[35] Ashhurst TM Marsh-Wakefield F Putri GH Spiteri AG Shinko D Read MN. Integration, Exploration, and Analysis of High-Dimensional Single-Cell Cytometry Data Using Spectre. Cytom Part A (2021) cyto.a.24350. doi: 10.1002/cyto.a.24350 33840138

[36] Van Gassen S Callebaut B Van Helden MJ Lambrecht BN Demeester P Dhaene T. FlowSOM: Using Self-Organizing Maps for Visualization and Interpretation of Cytometry Data. Cytom Part A (2015) 87(7):636–45. doi: 10.1002/cyto.a.22625 25573116

[37] Wragg KM Tan H-X Kristensen AB Nguyen-Robertson CV Kelleher AD Parsons MS. High CD26 and Low CD94 Expression Identifies an IL-23 Responsive Vδ2+ T Cell Subset With a MAIT Cell-Like Transcriptional Profile. Cell Rep (2020) 31:107773. doi: 10.1016/j.celrep.2020.107773 32553157

[38] van der Heiden M Björkander S Rahman Qazi K Bittmann J Hell L Jenmalm MC. Characterization of the γδ T-Cell Compartment During Infancy Reveals Clear Differences Between the Early Neonatal Period and 2 Years of Age. Immunol Cell Biol (2020) 98:79–87. doi: 10.1111/imcb.12303 31680329 PMC7003854

[39] De Rosa SC Andrus JP Perfetto SP Mantovani JJ Herzenberg LA Herzenberg LA. Ontogeny of γδ T Cells in Humans. J Immunol (2004) 172:1637–45. doi: 10.4049/jimmunol.172.3.1637 14734745

[40] Déchanet J Merville P Bergé F Bone-Mane G Taupin JL Michel P. Major Expansion of γδ T Lymphocytes Following Cytomegalovirus Infection in Kidney Allograft Recipients. J Infect Dis (1999) 179:1–8. doi: 10.1086/314568 9841815

[41] Knight A Madrigal AJ Grace S Sivakumaran J Kottaridis P Mackinnon S. The Role of Vδ2-Negative γδ T Cells During Cytomegalovirus Reactivation in Recipients of Allogeneic Stem Cell Transplantation. Blood (2010) 116:2164–72. doi: 10.1182/blood-2010-01-255166 20576814

[42] Rossol R Dobmeyer JM Dobmeyer TS Klein SA Rossol S Wesch D. Increase in Vdelta1+ Gammadelta T Cells in the Peripheral Blood and Bone Marrow as a Selective Feature of HIV-1 But Not Other Virus Infections. Br J Haematol (1998) 100:728–34. doi: 10.1046/j.1365-2141.1998.00630.x 9531341

[43] Boullier S Cochet M Poccia F Gougeon ML. CDR3-Independent Gamma Delta V Delta 1+ T Cell Expansion in the Peripheral Blood of HIV-Infected Persons. J Immunol (1995) 154:1418–31. 7822807

[44] Fujishima N Hirokawa M Fujishima M Yamashita J Saitoh H Ichikawa Y. Skewed T Cell Receptor Repertoire of Vdelta1(+) Gammadelta T Lymphocytes After Human Allogeneic Haematopoietic Stem Cell Transplantation and the Potential Role for Epstein-Barr Virus-Infected B Cells in Clonal Restriction. Clin Exp Immunol (2007) 149:70–9. doi: 10.1111/j.1365-2249.2007.03388.x PMC1942033 17425654

[45] Couzi L Pitard V Sicard X Garrigue I Hawchar O Merville P. Antibody-Dependent Anti-Cytomegalovirus Activity of Human γδ T Cells Expressing CD16 (Fcγriiia). Blood (2012) 119:1418–27. doi: 10.1182/blood-2011-06-363655 22180442

[46] Ravens S Hengst J Schlapphoff V Deterding K Dhingra A Schultze-Florey C. Human γδ T Cell Receptor Repertoires in Peripheral Blood Remain Stable Despite Clearance of Persistent Hepatitis C Virus Infection by Direct-Acting Antiviral Drug Therapy. Front Immunol (2018) 9:510. doi: 10.3389/fimmu.2018.00510 29616028 PMC5864898

