# Supplementary figures and images for: Evidence for an Adult-Like Type 1-Immunity Phenotype of Vδ1, Vδ2 and Vδ3 T Cells in Ghanaian Children With Repeated Exposure to Malaria
Source: Front Immunol. 2022 Feb 17;13:807765. doi: 10.3389/fimmu.2022.807765 (PMC8891705; doi:10.3389/fimmu.2022.807765)

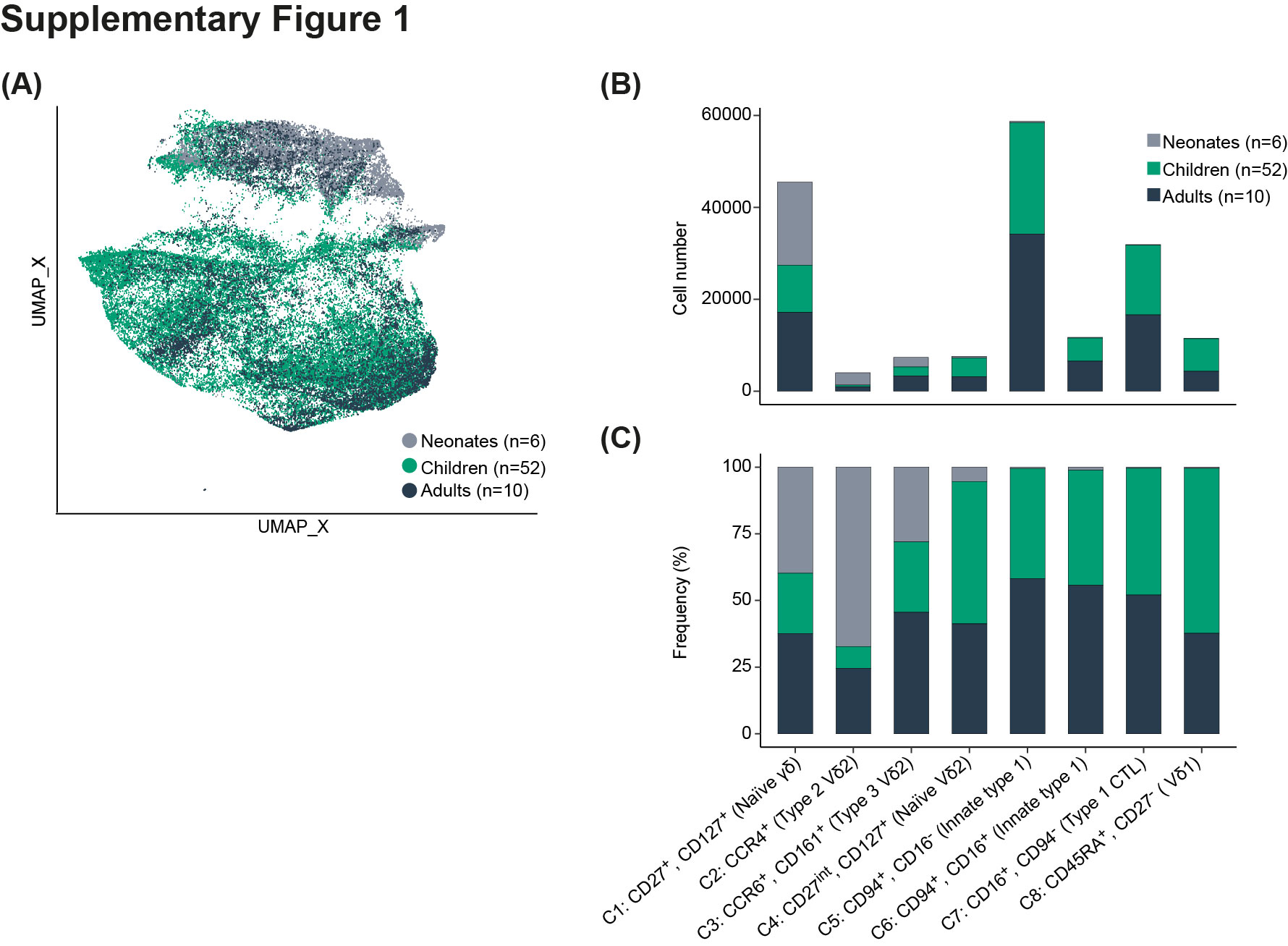

Supplement: Supplementary Figure 1 — Contribution to the identified clusters by age group. (A) Single cells visualized on UMAP color-code by age group in newborns (cord blood, n=6), children (n=52) and adults (n=10). (B) γδ T cell count contribution of newborns (cord blood, n=6), children (n=52), and adults (n=10) to each identified cluster in cell count and (C) frequency. [file Image_1.jpeg]

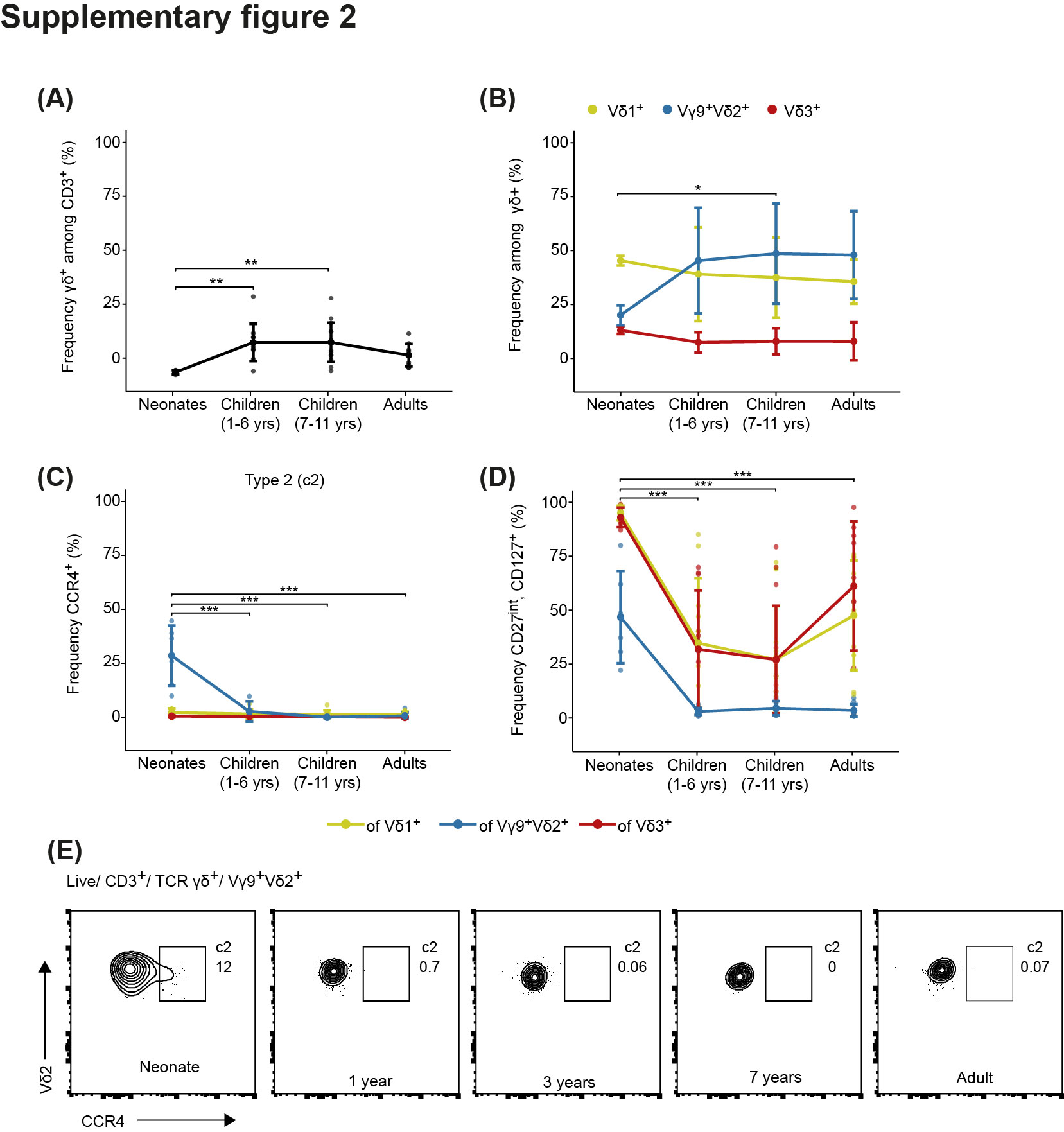

Supplement: Supplementary Figure 2 — Age-dependent distribution of γδ T cell subsets. (A) Frequencies of γδ T cells in newborns (cord blood, n=6), young children (1-6 year old, n=12), older children (7-11 years old, n=15), and adults (n=10) without malaria. (B) The frequency of Vδ1+, Vγ9+Vδ2+ or Vδ3+ of CD3+ γδ T cells per age group in malaria-free donors. (C) Frequencies of CCR4+ and (D) CD27int/CD127+ of the total Vδ1+, Vγ9+Vδ2+ or Vδ3+ T cells. Data was analysed by ANOVA and Turkey post-hoc test, figure C shows the results of Vγ9+Vδ2+ T cell comparisons. Error bars indicate mean + SD. *p < 0.05, **p < 0.01, ***p < 0.001. (E) Flow cytometric plot CCR4 on Vγ9+Vδ2+ T cells in representative samples from a neonate, children without malaria aged 1, 2 and 7 years and one adult. [file Image_2.jpeg]

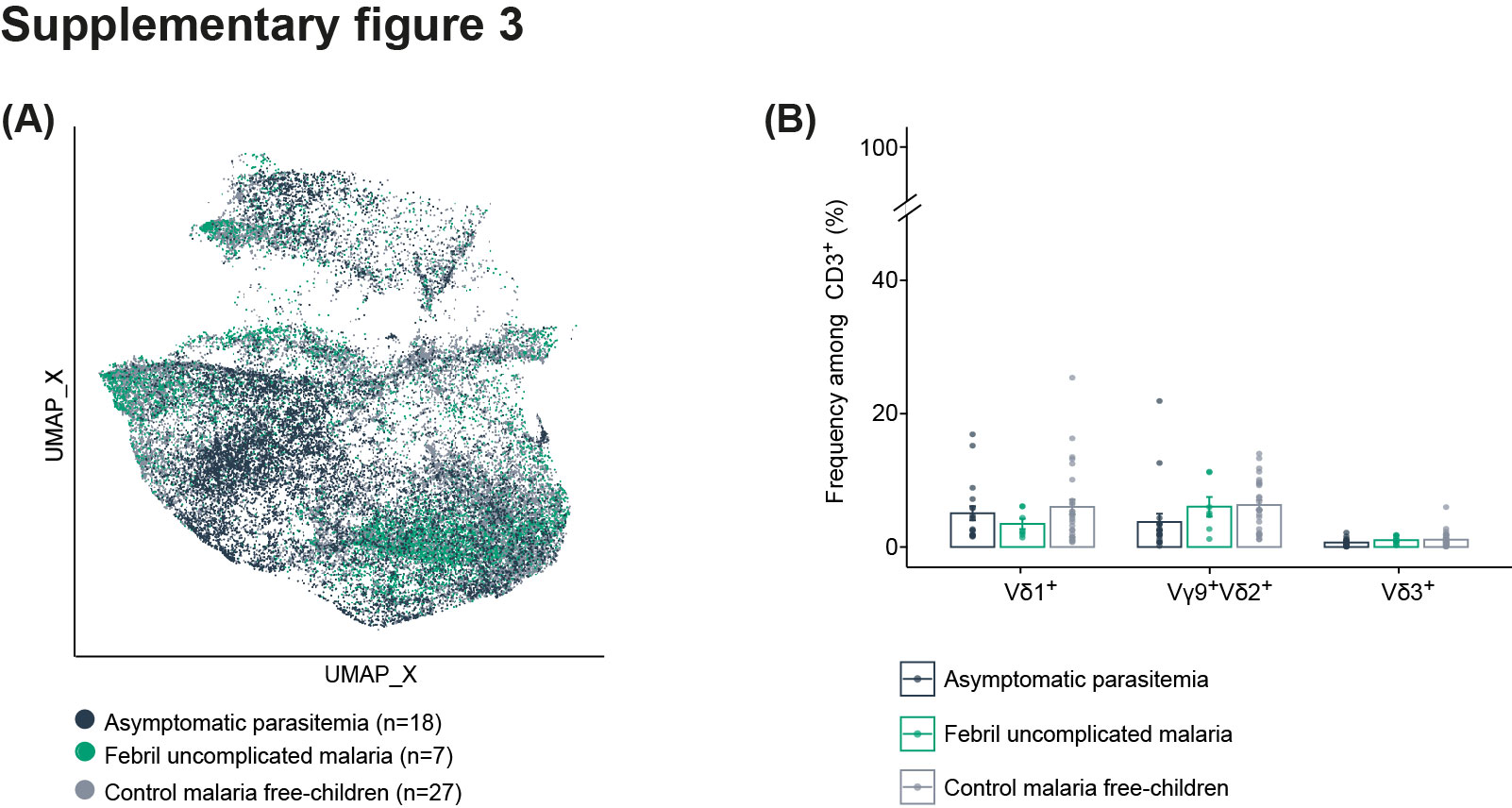

Supplement: Supplementary Figure 3 — γδ T cell effectors in malaria-free and malaria-positive children. (A) Single cells visualized on UMAP color- code by malaria status in either asymptomatic parasitemia (n=18), febrile uncomplicated malaria (n=7) or control malaria free-children (n=27). (B) Comparison of Vδ1+, Vγ9+Vδ2+ or Vδ3+ T cell frequencies of CD3+ T cells according to malaria status of children. [file Image_3.jpeg]
